# Association between Lipid Accumulation Product and Hirsutism in Patients with Polycystic Ovary Syndrome

**DOI:** 10.1055/s-0036-1571423

**Published:** 2016-02

**Authors:** Flávia Ribeiro de Oliveira, Mariana Bicalho Rezende, Nícolas Figueiredo Faria, Tomás Ribeiro Gonçalves Dias, Walter Carlos Santos de Oliveira, Ana Luiza Lunardi Rocha, Ana Lúcia Cândido

**Affiliations:** 1 Faculdade de Saúde e Ecologia Humana – FASEH, Vespasiano, Brazil; Laboratory of Human Reproduction, Hospital das Clínicas, Universidade Federal de Minas Gerais – UFMG, Belo Horizonte, MG, Brazil; 2 Undergraduate Medical Course, FASEH, Vespasiano, MG, Brazil; 3 Department of Gynecology and Obstetrics, School of Medicine; Laboratory of Human Reproduction and Hyperandrogenism Outpatient Clinic, Hospital das Clínicas, UFMG, Belo Horizonte, MG, Brazil; 4 Department of Clinical Medicine, School of Medicine; Hyperandrogenism Outpatient Clinic, Hospital das Clínicas, UFMG, Belo Horizonte, MG, Brazil

**Keywords:** polycystic ovary syndrome, hirsutism, cardiovascular disease, insulin resistance, síndrome dos ovários policísticos, hirsutismo, doença cardiovascular, resistência à insulina

## Abstract

**Objective** Polycystic ovary syndrome (PCOS) is the most common endocrine metabolic disorder in women between menarche and menopause. Clinical hyperandrogenism is the most important diagnostic criterion of the syndrome, which manifests as hirsutism in 70% of cases. Hirsute carriers of PCOS have high cardiovascular risk. Lipid accumulation product (LAP) is an index for the evaluation of lipid accumulation in adults and the prediction of cardiovascular risk. The aim of this study was to evaluate the association between LAP and hirsutism in women with PCOS.

**Methods** This was a cross-sectional observational study of a secondary database, which included 263 patients who had visited the Hyperandrogenism Outpatient Clinic from November 2009 to July 2014. The exclusion criteria were patients without Ferriman-Gallwey index (FGI) and/or LAP data. We used the Rotterdam criteria for the diagnosis of PCOS. All patients underwent medical assessment followed by measurement and recording of anthropometric data and the laboratory tests for measurement of the following: thyroid-stimulating hormone, follicle-stimulating hormone, prolactin, total testosterone, sex hormone binding globulin, 17-α-hydroxyprogesterone (follicular phase), glycohemoglobin A1c, and basal insulin. In addition, the subjects underwent lipid profiling and oral glucose tolerance tests. Other laboratory measurements were determined according to clinical criteria. LAP and the homeostatic model assessment index (HOMA-IR) were calculated using the data obtained. We divided patients into two groups: the PCOS group with normal LAP (< 34.5) and the PCOS group with altered LAP (> 34.5) to compare the occurrence of hirsutism. For statistical analysis, we used SPSS Statistics for Windows® and Microsoft Excel programs, with descriptive (frequencies, percentages, means, and standard deviations) and comparative analyses (Student's *t* -test and Chi-square test). We considered relations significant when the p-value was ≤ 0.05.

**Results** LAP was high in most patients ( *n* = 177; 67.3%) and the FGI indicated that 58.5% of the patients ( *n* = 154) had hirsutism. The analysis by LAP quartiles showed a positive correlation ( *p* = 0.04) among patients with a high FGI and an upper quartile LAP (> 79.5) when compared with those with LAP < 29.0 (lower quartile).

**Conclusion** This study demonstrated an association between high LAP and hirsutism. The FGI could represent a simple and low-cost tool to infer an increased cardiovascular risk in women with PCOS.

## Resumo

**Objetivo** A síndrome dos ovários policísticos (SOP) é o distúrbio endócrino mais comum em mulheres entre a menarca e a menopausa. O hiperandrogenismo clínico é o critério diagnóstico mais importante da síndrome, que se manifesta como hirsutismo em 70% dos casos. Portadores de SOP hirsutas têm elevado risco cardiovascular. O *Lipid accumulation product* (LAP) é um índice para a avaliação da acumulação de lípidos nos adultos e é um preditor de risco cardiovascular. O objetivo deste estudo é avaliar a associação entre LAP e hirsutismo em portadoras da SOP.

**Métodos** Estudo observacional transversal de banco de dados secundário, que incluiu 263 pacientes do Ambulatório de Hiperandrogenismo no período de novembro de 2009 a julho de 2014. Foram excluídas pacientes sem o índice de Ferriman-Gallwey e/ou LAP. Foram utilizados como critérios diagnósticos da SOP os critérios de Rotterdam. Todas as pacientes foram submetidas à avaliação médica seguida da aferição e registro dos dados antropométricos e a realização dos seguintes exames laboratoriais: hormônio estimulante da tireoide (TSH), hormônio folículo estimulante (FSH), prolactina (PRL), Testosterona total, globulina ligadora dos hormônios sexuais (SHBG), 17-α-hidroxiprogesterona, (fase folicular), perfil lipídico, teste oral de tolerância à glicose, glico-hemoglobina a1C, insulina basal. Outras dosagens laboratoriais foram determinadas à critério clínico. O LAP e o HOMA-IR ( *homeostatic model assessment* ) foram calculados com os dados obtidos. As pacientes foram divididas em dois grupos: grupo das portadoras de SOP com LAP normal (LA *p* < 34,5) e grupo das portadoras de SOP com LAP alterado (LAP >34,5) para as comparações do hirsutismo. Para a análise estatística, foram utilizados os programas SPSS Statistics for Windows® e Microsoft Excel®, sendo feitas análises descritivas (frequências, percentuais, médias, desvios-padrão) e comparativas (t-Student e qui-quadrado). Foram consideradas relações significativas quando p-valor foi menor ou igual a 0,05.

**Resultados** O LAP foi elevado na maioria das pacientes ( *n* = 177; 67,3%) e o índice de Ferriman-Gallwey (IF) demonstrou que 58,5% das pacientes ( *n* = 154) eram hirsutas. A análise por quartis de LAP, demonstrou correlação positiva ( *p* = 0,04) entre pacientes com IF elevado e LAP no quartil superior (> 79,5) quando comparadas àquelas com LAP menor que 29,0 (quartil inferior).

**Conclusão** O estudo demonstrou associação do LAP elevado e hirsutismo. O escore de Ferriman-Gallwey poderia representar uma ferramenta simples e de baixo custo para inferir risco cardiovascular aumentado em portadoras da síndrome.

## Introduction

Polycystic ovary syndrome (PCOS) is the most common endocrine metabolic disorder in women between menarche and menopause. It may reach a prevalence > 18% according to the Rotterdam classification criterion. 1 2 3 This condition can manifest in four different phenotypes: classical phenotype (anovulation or oligo-ovulation with irregular menses, clinical and/or laboratory hyperandrogenism, and polycystic ovaries on ultrasound, US); ovulatory phenotype (hyperandrogenism and polycystic ovaries on US); non-hyperandrogenic phenotype (anovulation or oligo-ovulation and polycystic ovary on US); and the phenotype that includes hyperandrogenism and anovulation or oligo-ovulation, but with no changes on US. 2 4

Clinical hyperandrogenism is an important diagnostic criterion of the syndrome, manifested as hirsutism (70%), acne (20%), and androgenic alopecia (5%). Hirsutism is evaluated by using the modified Ferriman-Gallwey index (FGI). The FGI is considered a good instrument for evaluating hirsutism, even considering ethnic differences among patients. 5 6 7 In adult women, hirsutism, acne, and alopecia are good substitutes of biochemical hyperandrogenism and should be considered as indicators of excessive androgen production. During adolescence, only hirsutism should be considered as a surrogate of biochemical hyperandrogenism, as acne is very common and often reversible, and alopecia is uncommon and generally has other causes. 5 In this study, we considered only hirsutism as an indicator of hyperandrogenism.

Hirsute carriers of PCOS have a 3-fold risk of developing metabolic syndrome (MS) compared with those without hirsutism. 8 9 MS occurs in ~43% of PCOS carriers, causing up to a 7-fold elevation of cardiovascular risk. 8 9 10

Lipid accumulation product (LAP) is an index for the evaluation of lipid accumulation in adults and the prediction of cardiovascular risk. This index was proposed for the first time in 2005 in an epidemiological study with the database of the National Health and Nutrition Examination Survey (NHANES III). 11 In that study, LAP showed better accuracy than body mass index (BMI) in the evaluation of cardiovascular risk in adult Americans. This index reflects lipid accumulation in adults in a simple way, and may be calculated by using the formula {LAP = [AC (cm) – 58] × TGL (mmol/L)}, 11 where AC is the abdominal circumference and TGL is the level of fasting triglycerides. In addition, high LAP is associated with type 2 diabetes mellitus and high mortality rates due to heart failure in women with normal weight but with high cardiovascular risk. 12 13

Recent studies in patients with PCOS suggest that LAP can be used to predict cardiovascular risk. 3 11 The homeostatic model assessment index (HOMA-IR) is more commonly used for the diagnosis of insulin resistance in patients with PCOS, and is calculated on the basis of the insulin and fasting glycaemia levels. 14 LAP showed greater accuracy than the HOMA-IR for the diagnosis of insulin resistance and glucose intolerance. 13 It is worth mentioning that subclinical damage in the organs associated with glucose intolerance is present before the onset of diabetes. 13 A patient with PCOS is considered a carrier of insulin resistance if LAP is > 34.5. 15

Thus, the objective of this study was to evaluate whether PCOS patients with increased LAP have a higher prevalence of hirsutism (FGI ≥ 8) than those with PCOS and normal LAP.

## Methods

This was an observational, cross-sectional study of a secondary database with 410 patients at the Hyperandrogenism Outpatient Clinic of the Hospital das Clínicas of the Universidade Federal de Minas Gerais. We obtained the data during the care of patients with PCOS in that service from November 2009 to July 2014. The inclusion criteria were as follows: patients with PCOS (Rotterdam criteria) less than 40 years old; those who had completed at least 2 years after menarche at the time of evaluation; BMI ≥ 18.5 kg/m ^2^ and < 40 kg/m ^2^ ; patients who were not using medications that interfere with hormone and/or metabolic measurements for at least 3 months; and patients in whom metformin use had been suspended for 2 months. The exclusion criteria were patients with diabetes mellitus type 1 (or 2), androgen-secreting tumors, Cushing syndrome, congenital adrenal hyperplasia, hyperprolactinemia, or untreated thyroid disorders. Patients with no data of abdominal circumference or triglyceride levels recorded in the database were excluded from the calculation of LAP. Finally, 263 patients were selected for the study.

The Rotterdam criteria were used for the diagnosis of PCOS, that is, the presence of two out of the following three findings: irregular menstrual cycles, clinical and/or laboratory hyperandrogenism, and presence of micro-polycystic ovaries upon US examination, after exclusion of other causes. All patients underwent medical assessment followed by measurement and recording of anthropometric data (weight, height, BMI, waist circumference, and blood pressure). They routinely underwent laboratory tests for measurements of the following: thyroid-stimulating hormone, follicle-stimulating hormone, prolactin, total testosterone, sex hormone-binding globulin, 17-α-hydroxyprogesterone (follicular phase), glycohemoglobin A1c, and basal insulin and underwent lipid profiling and oral glucose tolerance tests. Other laboratory measurements were determined according to clinical criteria. LAP and the HOMA-IR were calculated using the data obtained, as previously described.

We divided the patients into two groups: the PCOS group with normal LAP (< 34.5) and the PCOS group with altered LAP (> 34.5) to compare the occurrence of hirsutism. We considered clinical hyperandrogenism in the presence of hirsutism (a score of FGI ≥ 8 on physical examination) according to the modified criteria of Ferriman-Gallwey or the presence of moderate/severe acne (types 3 and 4) or androgenic alopecia.

In the second analysis, we divided patients per quartile, and the lower quartile (LAP < 29.0) was compared with the upper quartile (LAP > 79.5), by using the same cutoff point of the FGI.

In addition to the descriptive analyses (determination of frequencies, percentages, means, and standard deviation), we performed comparative statistical analyses. Among the comparative analyses, we used the Student's *t* -test to compare the means of the two independent groups and the chi-square test to compare categorical variables, in addition to the stratification of the data into quartiles, presented in a box-plot format. The SPSS Statistics for Windows® (IBM, Version 20.0. Armonk, NY) was used for the statistical analysis and the construction of the box-plots. Statistical significance was considered for a p-value ≤ 0.05.

The authors declare no conflict of interest in conducting this study.

## Results

Patients with PCOS and altered LAP had a lower mean value of high-density lipoprotein (HDL)-cholesterol (43.5 ± 9.5 mg/dL versus 50.7 ± 11.1 mg/dL; *p* = 0.0001), a higher average BMI (33.5 ± 5.8 versus 26.2 ± 4.9; *p* = 0.0001), and more criteria for the diagnosis of MS (2 ± 1 criteria versus 1 ± 1 criteria; *p* = 0.0001) than patients with PCOS and normal LAP. Table 1 summarizes the clinical, hormonal, and metabolic profiles in the two groups of patients (altered LAP versus normal LAP).

**Table 1:** Clinical, hormonal, and metabolic profile by groups (normal × altered LAP)

Parameters	Altered LAP	Normal LAP	p-value
Age (years)	33 ± 6	33 ± 5	0.638
Weight (kg)	87.7 ± 16.7	68.4 ± 15.5	0.0001
Height (cm)	1.62 ± 0.07	1.61 ± 0.08	0.552
BMI	33.5 ± 5.8	26.2 ± 4.9	0.0001
AC (cm)	104.4 ± 12.7	85.8 ± 10.9	0.0001
HDL (mg/dL) 2	43.5 ± 9.5	50.7 ± 11.1	0.0001
Fasting glucose (mg/dL)	88.3 ± 14	85.2 ± 14.9	0.099
TGL (mg/dL) 2	150.4 ± 66.1	74.1 ± 25.2	0.0001
Insulin (μIU/mL)	17.8 ± 19.5	10.1 ± 10.1	0.001
HOMA-IR index 2	3.9 ± 4.8	2.1 ± 2.4	0.002
Number of MS criteria	2 ± 1	1 ± 1	0.0001

Abbreviation: AC, abdominal circumference; BMI, body mass index; HDL, high-density lipoprotein; HOMA-IR, homeostatic model assessment of insulin resistance; LAP, lipid accumulation product; MS, metabolic syndrome; TGL, triglycerides.

We observed that most patients analyzed (63.7%; *n* = 177) had high LAP (≥ 34.5). The same was observed in relation to the measures of the FGI, i.e., most patients ( *n* = 154, 58.6%) had hirsutism.

**Fig. 1:**
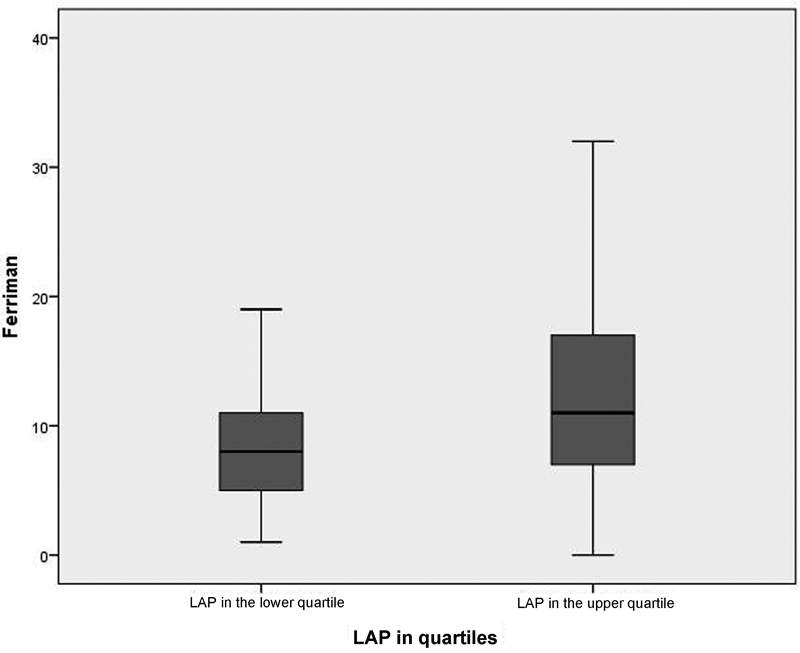
Box-plot comparing the Ferriman index with lipid accumulation product (LAP) in the upper and lower quartiles ( *p* = 0.049).

When analyzing the relation of the FGI by groups of LAP, we observed that in both groups of patients, there was a higher incidence of altered FGI (≥ 8), with an incidence of 52.3% for the normal LAP group (< 34.5) and 61.6% for the altered LAP group (≥ 34.5), without a statistically significant difference ( Fig. 1 ).

The lower (Q1 = 29.0) and upper (Q3 = 79.5) quartiles showed significant differences in LAP ( *p* = 0.04). This statistical relation was also confirmed using odds ratios that demonstrated that women with LAP > 79.5 (upper quartile) had a 2-fold higher association with the altered FGI.

## Discussion

Most patients in the study had high LAP (≥34.5) and hirsutism. Patients with a normal FGI had LAP in the lower quartile (60.8%). On the other hand, most patients with altered FGI had LAP in the upper quartile (56.8%), indicating that hirsutism is associated with insulin resistance and cardiovascular risk, corroborating published data. 12 13 In this context, it can be inferred that women with PCOS and hirsutism will have a greater chance of presenting an altered LAP index, indicating that this group of patients will present a higher risk of metabolic alterations. On the other hand, patients with the non-hyperandrogenic PCOS phenotype (normal FGI) present as a metabolically similar group to patients without PCOS. 14 Patients with PCOS and hirsutism have been reported to have an increased risk of MS. 8 9 10 16 The progressive accumulation of fat, mainly in the abdominal region, is characterized by an increase in insulin resistance. 13 LAP reflects both the deposition of visceral fat and the increase of lipolytic activity within adipose tissue. 13 The observation that patients with PCOS and hirsutism would also present an altered LAP index not only confirms the greater risk of MS but also allows for an early diagnosis of metabolic changes.

PCOS patients with an altered LAP index had lower mean HDL-cholesterol levels, higher mean BMI and HOMA-IR, and a greater chance of developing MS, reinforcing the finding previously described in the literature that LAP is a good predictor of cardiovascular risk. A recent meta-analysis showed that women with PCOS have lower levels of HDL-cholesterol, regardless of BMI. 17 Obesity has an estimated mean prevalence of 49% in PCOS patients, 18 ranging from 12.5% to 100%, 2 3 19 and its presence can aggravate metabolic and reproductive disorders associated with PCOS, 19 including dyslipidemia and MS; the prevalence of MS varies according to the clinical phenotypes of PCOS. 20

The groups evaluated in the study were relatively comparable and presented similar mean age and height. The hormonal and metabolic profile differed between groups, as expected, as the LAP is known to be associated with MS.

One of the main limitations of this study was that a secondary database was used as the data source obtained from the medical records of a single clinical center and the information contained therein was dependent on their correct completion.

The present study results showed that hirsutism correlates with an altered LAP index. This relation has clinical relevance because hirsutism, which is known to have a correlation with cardiovascular risk, is also related with an altered LAP index, indicating that patients with this condition have a higher risk of insulin resistance and cardiovascular disease. Hirsute women with PCOS should be perceived as having a potentially higher metabolic risk, a higher probability of insulin resistance, and other relevant comorbidities. The modified Ferriman-Gallwey scale could be a simple, low-cost, and useful way to infer an increased cardiovascular risk in patients with PCOS.
